# Dual-task costs in speed tasks: a comparison between elite ice hockey, open-skill and closed-skill sports athletes

**DOI:** 10.3389/fpsyg.2024.1357312

**Published:** 2024-07-15

**Authors:** Mark Brinkbäumer, Christian Kupper, Lukas Reichert, Karen Zentgraf

**Affiliations:** Department of Movement and Exercise Science, Institute for Sport Sciences, Goethe University, Frankfurt, Germany

**Keywords:** dual-task, dual-task cost, cognitive-motor interference, elite athletes, high performance

## Abstract

**Introduction:**

Ice hockey is a high pace sports game that requires players to integrate multiple skills. Players face perceptive, cognitive, and motor tasks concurrently; hence, players are regularly exposed to dual- or multi-task demands. Dual-tasking has been shown to lead to decreased performance in one or both performed tasks. The degree of performance reductions might be modulated by the exhaustion of cognitive resources. Literature on dual-task paradigms that combine sport-relevant elements is scarce. Therefore, a novel paradigm combining cyclical speed of the lower extremities and concurrent visuo-verbal speed reading was tested and validated. Additionally, to understand the nature of dual-task costs, the relationship between these costs and cognitive performance was assessed. We hypothesized occurrence of dual-task costs in all athletes without relationship to single task performance. Differences in dual-task cost were expected between open-skill and closed-skill sports, as well as differing expertise levels. Level of cognitive function was expected to explain some variance in dual-task cost.

**Methods:**

A total of 322 elite athletes (120 ice hockey, 165 other team sports, 37 closed-skill sports) participated in this study. Each athlete performed a tapping task, a visuo-verbal speed-reading task, and both tasks simultaneously. All ice hockey athletes performed additional cognitive tests assessing processing speed, spatial working memory, sustained attention, two choice reaction time, and motor inhibition.

**Results:**

The results of paired-sample t-tests confirmed significant dual-task costs for all sport groups (*p* < 0.001). Single-task performance and dual-task costs correlated weakly in a positive direction. A one-way ANOVA revealed significantly greater costs in closed-skill sports athletes than in ice hockey and other sports athletes. No significant differences in dual-task costs were found between teams of differing expertise levels. Lastly, no significant regression model was found to predict dual-task costs from cognitive test performance.

**Discussion:**

Our study suggests that this novel dual-task paradigm was successful in inducing dual-task costs for all elite athletes. Since it distinguishes between closed-skill and open-skill sports athletes, it might be a valuable diagnostic tool for performance and for talent development of open-skill athletes. Dual-task costs could not be relevantly predicted via cognitive performance measures, questioning cognitive resource theories as an explanation for dual-task costs.

## 1 Introduction

Ice hockey is a high-pace contact sports game in which players perform repeated bouts of high-intensity action. Players are rotated in shifts of 30–80 s over four quarters of 15 min (Burr et al., 2008; Bond et al., 2018; Vigh-Larsen et al., 2019). During the entire game, players are in action on ice for 15–25 min (Roczniok et al., 2016). Players must be physically fast and well-conditioned for a wide range of ice hockey actions, such as cuts, turns, weave agility, decelerations, accelerations, and collisions (Novák et al., 2019). Especially considering the evolution of the game towards increased game speed (Westerlund and Summanen, 2001; Thomas, 2006), there seems to be a striking need for fast players. A nine-year trend study by Bielmann et al. (2022) provides evidence that players are indeed improving their physical speed, at least in their sample of male U18 Swiss national ice hockey players. Moreover, successful performance requires the players to integrate multiple skills from different domains, i.e., locomotion while passing or shooting (Fait et al., 2011). This integration in game-like behaviour has substantial information processing demands, especially on the perceptual and cognitive side (Broadbent et al., 2014).

In the increasingly dynamic field of modern sports, the interplay of physical and cognitive abilities seems to be essential (Ghasemzadeh and Saadat, 2023). For optimal performance, both seem to be intricately connected (Navabinejad and Rostami, 2023). Perceptual-cognitive function and skill are factors associated with superior sport performance (Scharfen and Memmert, 2019). Cognitive performance can be distinguished into domain-specific skills or domain-general functions (Kalén et al., 2021). The “expert performance approach” (Ericsson, 2003) has consistently shown that experts outperform novices on domain-specific tests that require visual scanning, prediction, spatial memory, and decision making (Janelle and Hillman, 2003; Mann et al., 2007). Though domain-specific tests are ecologically valid and can shed some light on differences between athletes of varying expertise levels (Ericsson, 2003), test results are heavily influenced by athletes’ procedural and declarative knowledge (Voss et al., 2010). This makes comparisons across sports hardly possible (Vestberg et al., 2012). The “cognitive component approach” assesses domain-general functions (Voss et al., 2010). Several studies found that players with greater expertise show superior performance in cognitive functions such as processing speed (Chaddock et al., 2011), working memory (Vaughan and Laborde, 2021), hand and foot motor inhibition (Heppe and Zentgraf, 2019), multiple object tracking (MOT) (Qiu et al., 2018), executive functions (Vestberg et al., 2012; Verburgh et al., 2014) and attention (Moratal et al., 2020) compared to less experienced players. Domain-general assessment allows comparisons across different sports (Mann et al., 2007; Voss et al., 2010; Wang et al., 2013; Krenn et al., 2018; Formenti et al., 2021; Koch and Krenn, 2021; Heilmann et al., 2022). According to their cognitive-perceptual demands, sports can be classified into open-skill and closed-skill sports (Wang et al., 2013; Gu et al., 2019; Zhu et al., 2020; Formenti et al., 2021; Koch and Krenn, 2021; Yongtawee et al., 2021; Heilmann et al., 2022). Open-skill sports take place in a dynamic environment, where athletes are externally paced and must constantly adjust to teammates and opponents. Ice hockey would be an example of open-skill sport. Closed-skill sports are more predictable and routine, allowing a greater extent of internal pacing by the participating athletes. Gymnastics is an example of closed-skill sport. In a meta-analysis, Zhu et al. (2020) found that closed-skill sport athletes show inferior performance in executive functions compared to open-skill sport athletes, when looking at cross-sectional studies. Data from interventional studies does, however, not support the advantage of open-skill sport for executive functions (Zhu et al., 2020).

There is little work on cognitive abilities of ice hockey players. In Faubert and Sidebottom (2012) discussed an approach to practice high-level athletes’ perceptual-cognitive skills via a three-dimensional MOT system. Several professional team sport athletes, including players from two National Hockey League (NHL) teams, were part of the sample. All professional players increased their perceptual-cognitive performance in the MOT task with training, with no differences between sports. The position in which players completed the test however seemed to influence the performance. One NHL team, which performed the test standing, performed worse than all other teams tested sitting.

Zhang et al. (2021) investigated the relationship between brain activity and attentional performance in a MOT-test among ice-hockey players. They found that elite players outperformed intermediate players in tracking accuracy and demonstrated higher individual alpha peak frequency, an electroencephalogram variable associated with attention and working memory. Furthermore, Lundgren et al. (2016) compared elite ice hockey players to a standardized sample consisting of 1750 nonclinical individuals ranging in age from 8 to 89 years. They found that the athletes scored significantly higher on design fluency, a measure of executive function from the Delis-Kaplan Executive Function System battery (Baldo et al., 2001; Delis et al., 2001). Additionally, they report a robust correlation between on-ice performance and trail making test scores, however, no differences between higher-and lower-league players were found. Lastly, James (2023) investigated the differences in executive functions between different divisions of Swedish ice hockey players. Coherent with Lundgren et al. (2016), no differences between divisions were found for inhibition and updating. Unexpectedly, shifting favoured lower division athletes. In general, more research on cognitive abilities of ice hockey athletes is needed.

Since team sport athletes in general face motor and perceptual-cognitive demands simultaneously, they are regularly exposed to dual-or multitasking scenarios (Moreira et al., 2021). Multitasking can be defined as the timely overlap of cognitive and motor processes when performing two (or more) tasks (Koch et al., 2018). This is typically accompanied by a decrease in performance of one or more tasks (dual−/multi-task cost) (Beurskens et al., 2015, 2016a,b; Plummer et al., 2015; Belghali et al., 2017; Solomito et al., 2018). In their review, Moreira et al. (2021) found that despite regularly being exposed to time-constrained situations and multitasking, athletes showed acute motor and cognitive performance costs in dual-task (DT) situations. In one of the included studies, Qiu et al. (2018) investigated the effect of varying expertise levels of basketball players on MOT task performance and found that experts demonstrated better performance than amateurs, who in turn performed better than novices. These results are in line with the results of Amico and Schaefer (2022) who found that experts outperformed intermediate tennis players. This suggests that superior DT performance can be an indicator of skill level in open-skill sports. Regarding the aetiology of performance costs, Moreira et al. (2021) discuss that high working memory capacity could be a resource that enables superior DT performance. Additionally, Fleddermann et al. (2019) found that chronic DT exposure leads to improvements in measures of processing speed and sustained attention. Casella et al. (2022) also reported improvement in all measures of cognitive functioning and attention capacity after performing 10 weeks of cognitive-motor DT training in young soccer players. This highlights a possible relationship between cognitive abilities and executive functions and DT performance, yet the direction of impact is unclear.

In social and cognitive psychology, a lot of research has been performed on the topic of dual−/multi-tasking and plenty of theories try to explain the underlying processes (Koch et al., 2018). Early studies conceptualize information processing as structurally limited to a “single channel” (Broadbent, 1958). According to this view, it is not possible for two channels to be processed simultaneously. This processing bottleneck, all-or-nothing perspective or attention “filter” requires fast channel switching in a DT setting (Lachter et al., 2004). This account specifically relates to early processing steps of perception (Broadbent, 1958). According to Kahneman (1973), DT costs (DTC) can be ascribed to the division of attention, which he theorized to be a limited central processing resource. This division of attention concept fits nicely to the concept of graded sharing of a central resource, postulated by Pashler (1998). Pashler (1994) further states there is no central bottleneck on the perceptual level, but rather in response selection. Current evidence that shows shared coding processes of perception and action opposes this view and questions the sequential nature of information processing (Fischer and Plessow, 2015). However, the assumption of sequential information processing, especially with the locus-of-slack-logic seems to be a useful heuristic that advanced research in the area (Koch et al., 2018). Fischer and Plessow (2015) describe the response selection bottleneck as context-sensitive, optimizing for performance. Presumably, multitasking requires maintenance of a balance between minimizing between-task interference, by serial task processing, and minimizing mental effort, by allowing for more parallel processing. Navon (1984) advanced the concept of a central resource and stated that there is not one, but multiple, domain-specific resources. Wickens (2008) proposes a multiple resource theory with four dimensions: (1) stages of processing distinguish between perceptual and cognitive tasks and selection and execution of action. (2) codes of processing differentiate between spatial activity and verbal/linguistic activity. This dimension is expressed in perception, working memory and action. (3) modalities (of perception), recognizes different resources for auditory and visual perception. (4) visual channels indicate a difference between focal and ambient vision within visual resources. If two tasks draw from distinct resources, less performance losses are to be expected. Assuming a domain-general resource that is shared by all tasks, at least one of the tasks must suffer from decreased performance when they overlap temporally. Several studies seem to confirm that when a visual-manual and an auditory-vocal ask are performed concurrently, there are smaller costs than when an auditory-vocal and auditory-manual task are performed at the same time (Hazeltine et al., 2006; Stelzel et al., 2006; Göthe et al., 2016). These findings are in line with Wickens (2008) since the latter combination of tasks should produce overlap in the perceptual-auditory resource.

Wollesen et al. (2022) discuss an increase in DT efficiency through greater task automatization as a possible adaptation to DT exposure. Hence, it may be important to test cognitive-motor interference in paradigms that are similar and specific to the sport, regarding both motor and cognitive demands. However, only five of the studies included in the review of Moreira et al. (2021) had a sport-specific paradigm (Helm et al., 2016; Gutiérrez-Davila et al., 2017; Fleddermann et al., 2019; Laurin and Finez, 2020; Schaefer and Scornaienchi, 2020). One very important physical feature that is recurring in many team sports is rapid locomotion, i.e., speed of movement (Brown and Ferrigno, 2015). Hence, it could be promising to examine DT performance with a motor task focusing on speed. The lower-body tapping task has been employed to assess cyclical speed of athletes (Chaabouni et al., 2022) and might be an option as an easily implemented and sufficiently specific motor task for team sports. On the cognitive side, next to perception, communication seems to be a crucial aspect of sports (Ishak, 2017). Especially verbal communication seems to be important to give teammates increased opportunity to notice and act upon events in the complex, fast-paced, highly contingent environment of actual play (LeCouteur and Feo, 2011). Players must quickly recognize observed scenarios and verbalize them to teammates to aid in his/her own perception.

In summary, ice hockey, like other team sports, has high cognitive and motor demands, which occur simultaneously in a match. Concurrent cognitive and motor tasks usually result in performance decrements or cognitive-motor interference. Superior ability to handle these kinds of scenarios might distinguish experts from less skilled players. Nevertheless, empirical findings show that even elite athletes demonstrate decreased performance under DT scenarios. Possible explanations for this phenomenon might be underlying cognitive resources, lacking automatization of motor skill or an interference between the representations engaged by central operations. Research on DT paradigms focusing on speed in athletes is lacking, hence a novel paradigm involving a lower-body tapping task and visuo-verbal speed-reading task is proposed.

The aim of this study is to (1) test a new DT paradigm focusing on cyclical speed of the lower extremities and concurrent visuo-verbal speed reading in ice hockey players, (2) check if DTC arise independently of ST performance, (3) validate it against other open-skill and closed-skill sports (4) compare performance between different teams/age groups, (5) assess the relationship between DT performance and cognitive functions, to potentially identify underlying resources. The working hypotheses of this study are (1) the employed paradigm will be demanding enough to provoke significant performance costs in elite ice hockey players and other elite athletes, (2) DTC will not be correlated to ST performance, (3) there will be no differences in DTC between ice hockey players and other open-skill sports athletes, but closed-skill sports athletes will display significantly greater costs than both groups, (4) more experienced players will display less DTC than less experienced (men’s U20 < men’s U18 & women’s first team < women’s U18), (5) if DTC rely on cognitive resources, there should be a relationship between measures of cognitive functions and DTC.

## 2 Materials and methods

### 2.1 Subjects

The data was gathered during two projects by the German Federal Institute for Sport Science. Testing of the athletes ensued in the context of extensive performance diagnostics, which was performed during German national team training camps (with the exemption of the handball players). A total of 322 elite athletes participated. All confirmed mental and physical readiness and signed a consent form before participating. Only athletes with healthy vision or correction (glasses or contact lenses) were allowed to participate. One-hundred and twenty ice hockey athletes of the German national team (*w* = 40, *m* = 79), aged 18.66 ± 3.91 years, participated. Players were part of women’s A (first) team (*n* = 18), women’s U18 (*n* = 22), men’s U20 (*n* = 36) and men’s U18 (*n* = 44). Furthermore, 165 elite team sport athletes were part of the performance diagnostics. Seventy-one were volleyball athletes of the German national team (*w* = 32, *m* = 39), aged 20.02 ± 5.02 years, 68 were basketball athletes of the German 3 × 3 and 5 versus 5 national team (*w* = 29, *m* = 39), with an average age of 21.67 ± 4.66 years, 15 were male handball players of the highest German league, aged 22.67 ± 3.16 years and 12 (*w* = 5, *m* = 7) were table tennis players from the German national team, aged 28.10 ± 6.51 years. Lastly, 37 athletes from closed sports took part. Eleven were modern pentathletes from the German national team (*w* = 9, *m* = 2), aged 23.78 ± 5.53 years, and 28 were trampoline gymnasts from the German national team (*w* = 12, *m* = 14), aged 18.58 ± 4.18 years.

### 2.2 Procedure

**Figure 1:**
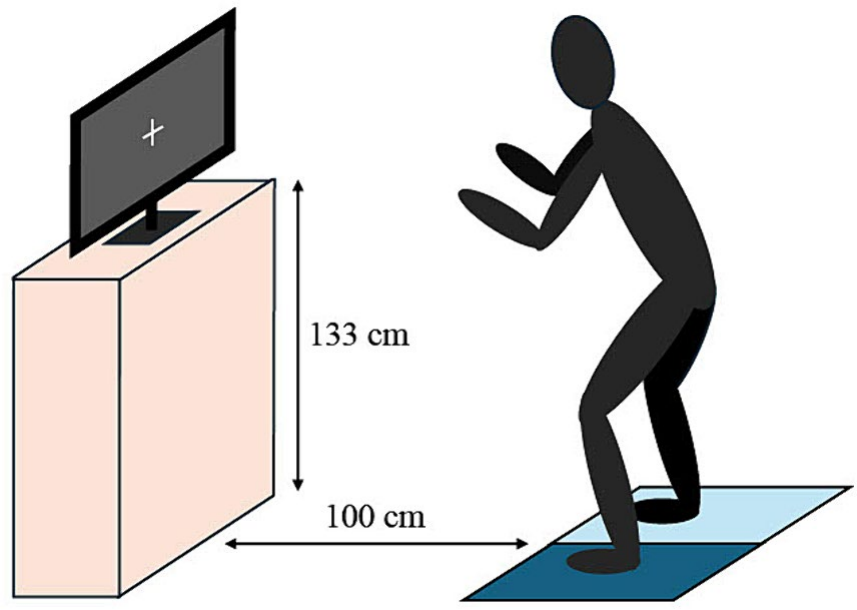
The experimental setup of the dual-task test paradigm.

All participants performed the following DT paradigm, which consisted of a motor single task (ST), cognitive ST, and a DT, which combined both ST. All subjects started with the motor ST, proceeded with the cognitive ST and finished with both at the same time as the DT. The experimental setup is illustrated in Figure 1. Cognitive tests were performed prior to DT testing.

### 2.3 Motor task

For the motor ST, tapping performance was measured via a contact mat (Sport Voss©). Subjects were instructed to alternately tap with the left and right leg onto the device as many times as possible, over a duration of 5 s. The test was started with an acoustic countdown and ended with final acoustic signal. Subjects started out of a standardized position with both feet on the mat with the knees slightly bent in a slight forward-leaning position. The arms were in front of the body and kept in position. The main parameter used was the maximal frequency (in Hz) of tapping, which was calculated as the highest frequency within 1 s at any time point. During the execution of the motor task, subjects were asked to keep the gaze on a cross (“+”) at a screen in front of them. The screen (Samsung, SyncMaster 2,494 HS, Suwon, South Korea) was positioned 100 cm in front of the contact mat at a height of 133 cm. This visual fixation was chosen to control the focus of visual attention across the ST and DT conditions. After performing a test trial, subjects performed two trials and an additional third if the discrepancy between trials was greater than 10%.

### 2.4 Cognitive task

**Figure 2:**
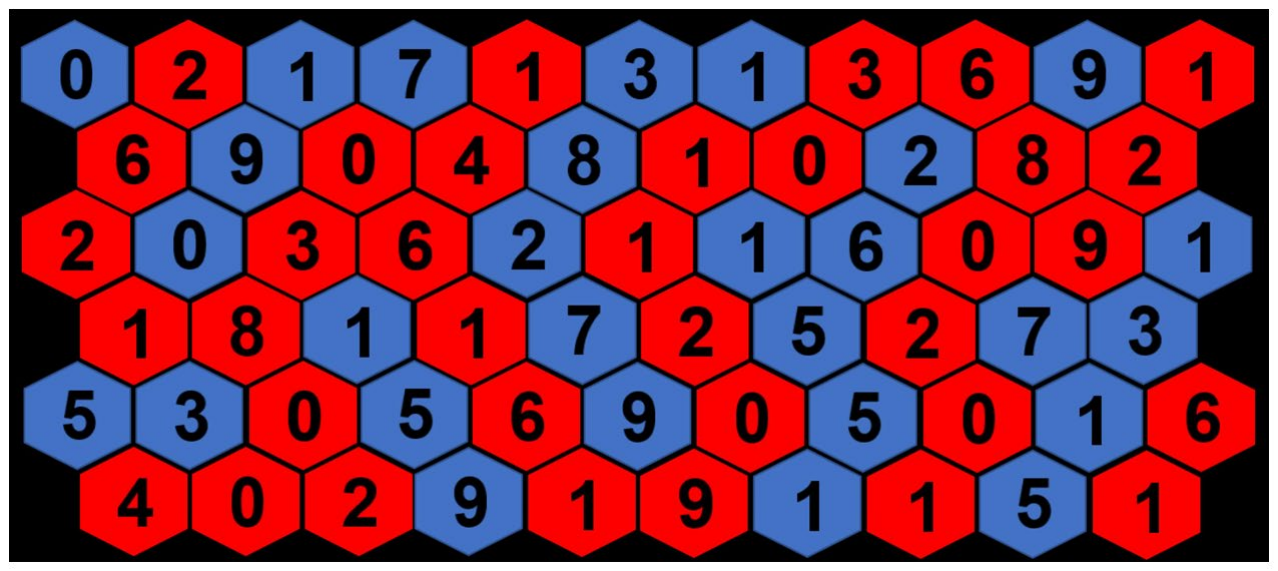
Exemplary visual speed-reading stimuli.

For the cognitive ST, subjects were instructed to stand on top of the contact mat in the same position as during the motor task. The acoustic start signal remained the same as in the motor task. Simultaneously to the start signal, subjects were presented with a honeycomb structure on the screen in front of them (Figure 2). Six rows with ten to eleven combs (a total of 63 combs) were presented. Every comb had a number between one and nine. Twenty-eight combs were colored in blue and the remaining in red. The stimuli were created and presented via Power Point (Microsoft, Washington, United States). Subjects had to read all numbers in blue combs as fast as possible, from left to right, top to bottom. Measurement stopped after all numbers were read. To ensure that subjects performed the cognitive task, the verbal answers were recorded using a recording mobile app. Reading time (in seconds) as well as errors were evaluated afterwards. Following a practice trial, subjects performed two trials.

### 2.5 Dual-task

In the DT condition, both tasks were performed simultaneously. The measurement started with the same acoustic signal as the ST. Subjects were instructed to tap as fast as possible while reading as fast and accurate as possible. They were explicitly told that both tasks were of the same importance, to minimize prioritization of any task. In contrast to the single motor task, tapping was not ended with an acoustic signal, but continued until all numbers were read. After performing a test trial, subjects performed two trials and an additional third if the discrepancy between trials was greater than 10% for the motor performance. Additionally, motor DTC was calculated as the difference between DT and ST performance.

### 2.6 Cognitive testing

Processing speed, sustained attention, spatial working memory, and motor inhibition performance was measured. Processing speed was assessed by the “Zahlen-Verbindungs-Test” (ZVT) (Oswald and Roth, 1987). In this paper-and-pencil test, participants must connect as many numbers as possible from 1 to 90 in the correct order within a time frame of 30 s. Overall, the test consisted of four test sheets. The dependent variable was the arithmetic mean of the accomplished items in the four sheets. The more numbers were connected correctly, the better the performance. The test was performed in the group test version according to the test manual (Oswald, 2016). The test has been shown to be a useful test with sufficient test reliability and validity (Rost and Hanses, 1993).

Sustained attention was measured by the d2-R test (Brickenkamp et al., 2010). This paper-and-pencil test measures attention and concentration ability under time pressure. The test consists of 14 lines with 47 characters per line with a randomized order of the letters “d” and “p.” Each letter is equipped with one, two, three, or four vertical stripes below or above the letter. The task of the participants is to mark the letter “d” with two stripes. All other characters are distractors, and participants are supposed to ignore these characters. For each line, participants have 20 s, and there is no break between the lines. The dependent variable is concentration performance (CP), which is calculated from the processed target objects and the errors. The higher the score, the better the performance. The test execution was performed in accordance with the test manual (Brickenkamp et al., 2010). Quality criteria for the d2-R test have been shown to be to be sufficiently met (Antretter et al., 2013).

Spatial working memory was measured using the forward and backward Corsi block test (CBT) (Schellig, 2011). This test has been established as a measure of spatial memory in both clinical and research contexts for several decades (Farrell Pagulayan et al., 2006). Testing ensued in isolation, individually on a laptop (Lenovo ThinkPad L460, Hongkong) using a CBT script, programmed on PsyToolkit (Stoet, 2010, 2017), supervised by a researcher. In the forward CBT, the participants are presented with nine pink, irregularly positioned blocks on a black screen. At the beginning of the test, two of these blocks light up yellow in a specific order. The participant must remember and replicate this order, i.e., the first item must be clicked first and the last item last. If the order is clicked correctly, the number of boxes lighting up increases by one. If the order is clicked incorrectly, the same number of boxes lights up. The test ends once the order is clicked incorrectly two consecutive times. In case the participant correctly clicks all nine boxes, the test also ends. The procedure for the backward CBT was the same as for the forward CBT, but the participants must remember the order and replicate it in reverse, i.e., the last presented item must be clicked first and the last presented item must be clicked first. Average number of correctly clicked items was recorded as outcome measure. Arce and McMullen (2021) evaluated the differences between physical and digital CBT and conclude that “most evidence today suggests that they are comparable.

Motor inhibition was measured using the stop signal reaction time (SSRT) test developed by Verbruggen and Logan (2008) with the modifications first introduced by Heppe and Zentgraf (2019). Participants were faced with a go-and a stop-condition, which they completed with hands only. The go-condition was employed to determine the choice reaction time. Participants are confronted with white arrows (left or right) and must press a pad with the corresponding hand. In 25% of the trials, the stop-condition is presented. In this condition, the white arrow turns blue after a variable stop signal delay (SSD) and the participants must inhibit the planned motor response. Stimuli are presented until an answer (press on the pad) is given. An adaptive staircase procedure is used for SSD to increase or decrease the task difficulty, around the individual threshold, and produce an inhibition success rate of about 50%. This entailed a decrease of SSD if an inhibition was successful and an increase of SSD if an inhibition was failed. To measure response inhibition an integration method was used (Heppe and Zentgraf, 2019). Two-choice reaction time (2CRT) was measured in the process of determining SSRT and included as another dependent variable. The test was performed in isolation, supervised by a researcher.

### 2.7 Statistical analysis

Data is reported in *M* ± *SD*. DTC were calculated as the difference between DT performance and ST performance. For statistical analysis SPSS Statistics Version 26 (IBM Corporation, Armonk, United States) was used. The level of significance was set at *p* < 0.05. The difference between ST and DT was assessed via paired sample t-tests to investigate the effect of performing both tasks simultaneously. In order to rule out a direct relationship between ST performance and DTC, a Pearson’s correlation was performed. To investigate relationship between sport type and DTC, group differences in DTC were explored with a one-way ANOVA for sport type (ice hockey, open-skill sports and closed-skill). A one-way ANOVA for national team (women’s first team, women’s U18, men’s U20, men’s U18) was performed to assess the differences between varying expertise levels and DTC. *ƞ^2^* was used as a measure of effect size. A multiple linear regression was calculated to investigate the relationship between the dependent variable DTC and the independent variables ZVT raw value, d2-R concentration performance, CBT span forward, CBT span backward, SSRT and 2CRT, in ice hockey athletes.

## 3 Results

### 3.1 Descriptive

**Figure 3:**
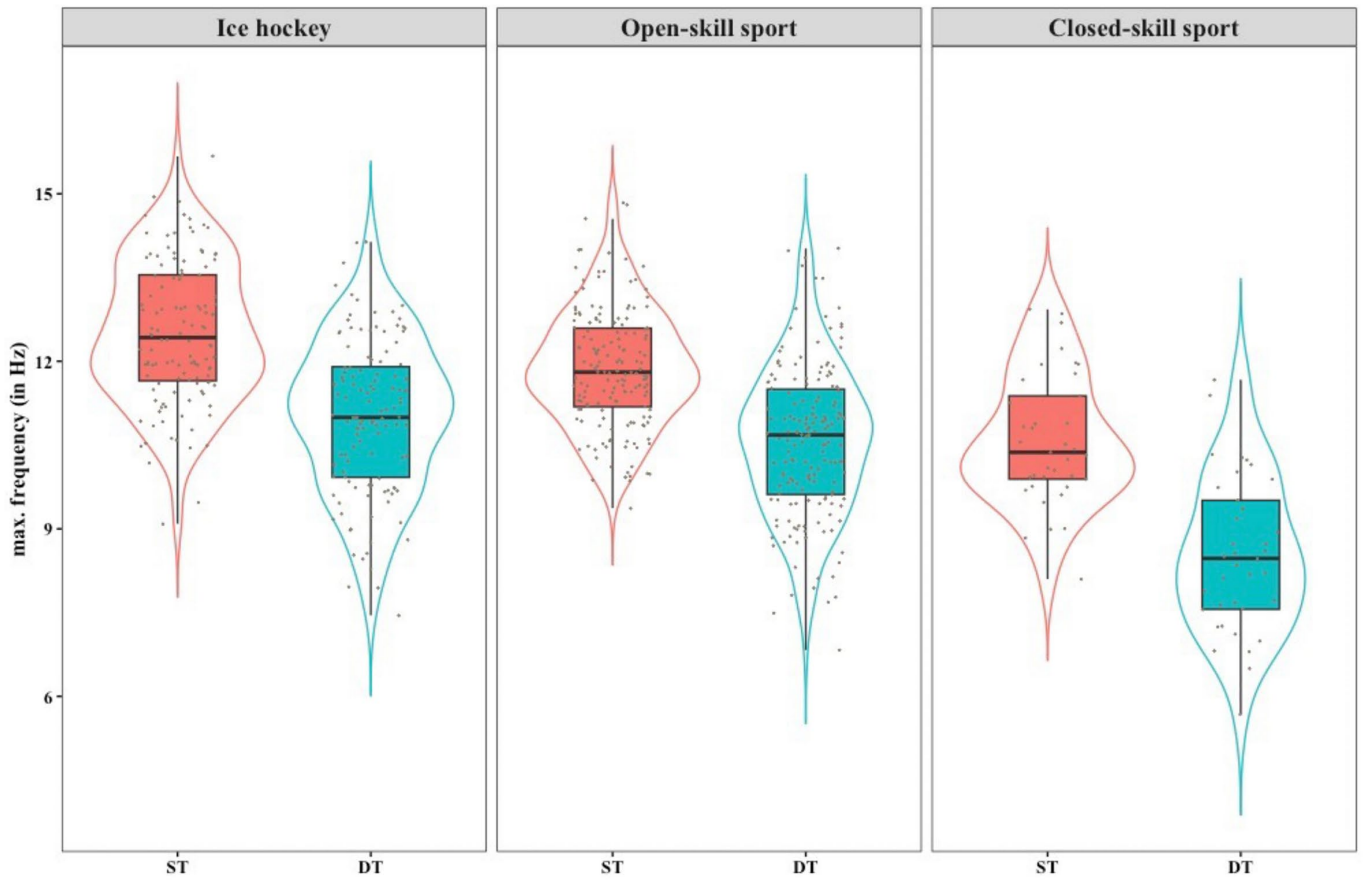
Overview of the ST and DT performance of ice hockey, open-skill and closed-skill sport athletes.

The mean ST performance was 12.51 Hz ± 1.27 for the ice hockey athletes, 11.85 Hz ± 1.04 for the other open-skill sports athletes and 10.58 Hz ± 1.17 for closed-skill sports athletes. The mean DT performance was 11.00 Hz ± 1.39 for the ice hockey athletes, 10.57 Hz ± 1.36 for the other open-skill sports athletes and 8.53 Hz ± 1.38 for closed-skill sports athletes. This resulted in DTC of 1.51 Hz ± 1.01 [−0.82, 4.61] for ice hockey athletes, 1.30 Hz ± 0.94 [−0.77, 5.35] for the other open-skill sport athletes and 2.05 Hz ± 1.11 [0.06, 4.59] for closed-skill sport athletes. Individual ST and DT performance for ice hockey, open-skill and closed-skill sport athletes are depicted in Figure 3.

### 3.2 ST and DTC

The Shapiro–Wilk’s test revealed normal distributions for ST and DT performance of all groups, ice hockey ST (*W* = 0.988, *p* = 0.408) & DT (*W* = 0.990, *p* = 0.503), open-skill sports ST (*W* = 0.994, *p* = 0.728) & DT (*W* = 0.994, *p* = 0.782) and closed-skill sports ST (*W* = 0.969, *p* = 0.377) & DT (*W* = 0.982, *p* = 790). Paired sample t-tests revealed a significantly lower performance in the DT condition for ice hockey *t*(119) = 16,367, *p* < 0.001, open-skill sports *t*(165) = 17.897, *p* < 0.001, and closed-skill sports *t*(36) = 11,223, *p* < 0.001. The Shapiro Wilk’s test indicated a normal distribution for ST performance (*W* = 0.988, *p* = 0.408) and DTC (*W* = 0.984, *p* = 0.153). ST and DTC were found to be significantly positively correlated *r*(120) = 0.273, *p* = 0.003.

### 3.3 DTC and sport types

**Figure 4:**
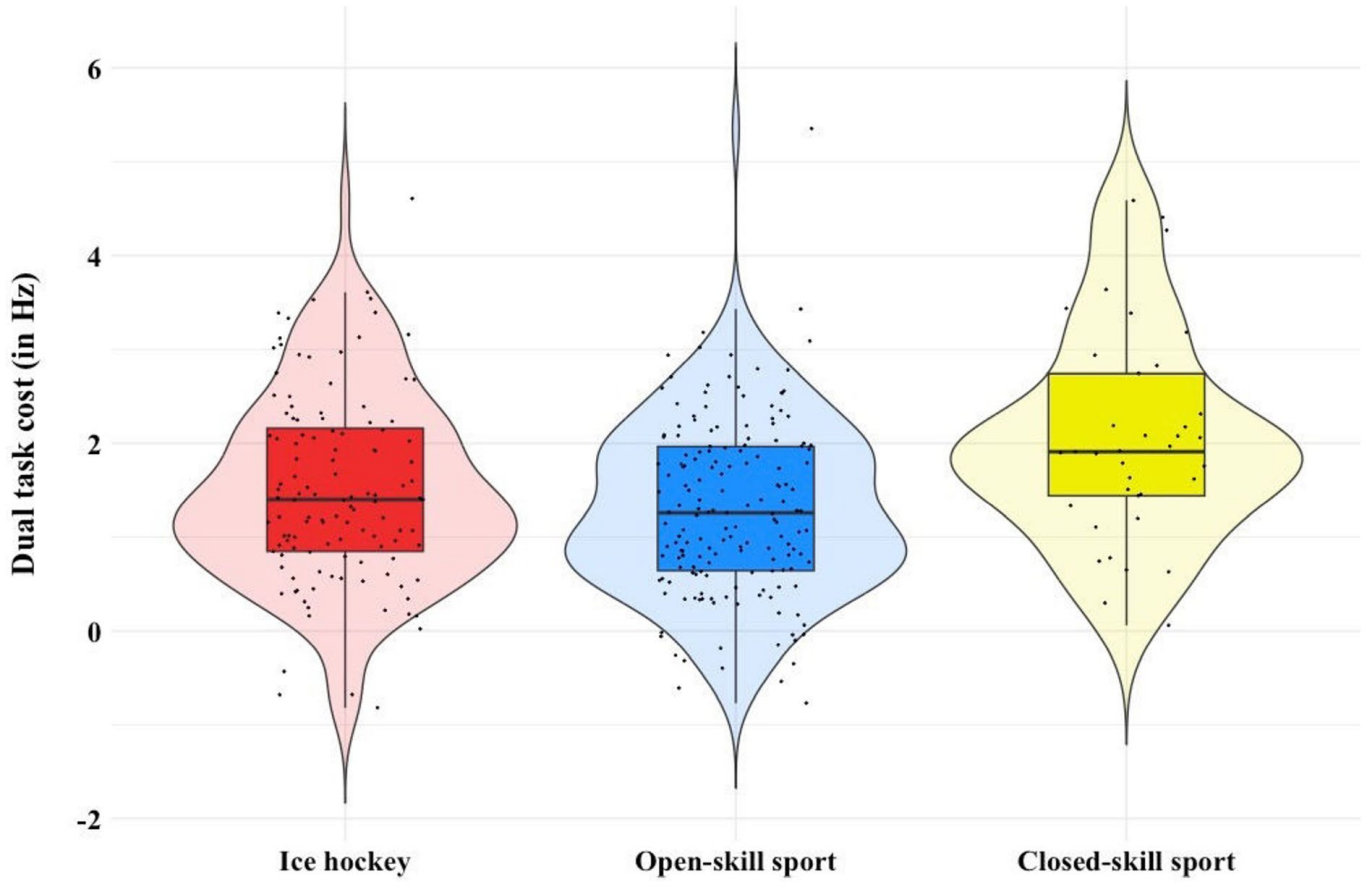
Comparison of dual-task costs between ice hockey, open-skill and closed-skill sports.

Figure 4 illustrates the difference in DTC between sport types. The Shapiro Wilk’s test confirmed normality of the data for DTC in ice hockey players (*W* = 0.984, *p* = 0.153), other open-skill sports athletes (*W* = 0.99, *p* = 0.296) and closed-skill sports athletes (*W* = 0.958, *p* = 0.170). Homogeneity of variance was confirmed, as assessed by Levene’s test for equality of variances (*p* = 0.443). A box plot indicated one outlier, which was checked, but kept in the data because it was a genuine value. The test revealed statistically significant differences in DTC between groups, *F*(2, 319) = 10.064, *p* < 0.001. There was no statistically significant difference between ice hockey and open-skill sports (*p* = 0.111). However, there was a mean decrease of 0.54 Hz, 95% *CI* [0.05, 1.03] in DTC from closed-skill sports (2.05 ± 1.11 Hz) to ice hockey (1.51 ± 1.01 Hz), which was statistically significant (*p* = 0.009). A comparison of closed-skill sports to open-skill sports (1.28 ± 0.89 Hz) showed a mean decrease in DTC of 0.77 Hz 95% *CI* [0.30, 1.25], which was also statistically significant (*p* < 0.001).

### 3.4 DTC and expertise

**Figure 5:**
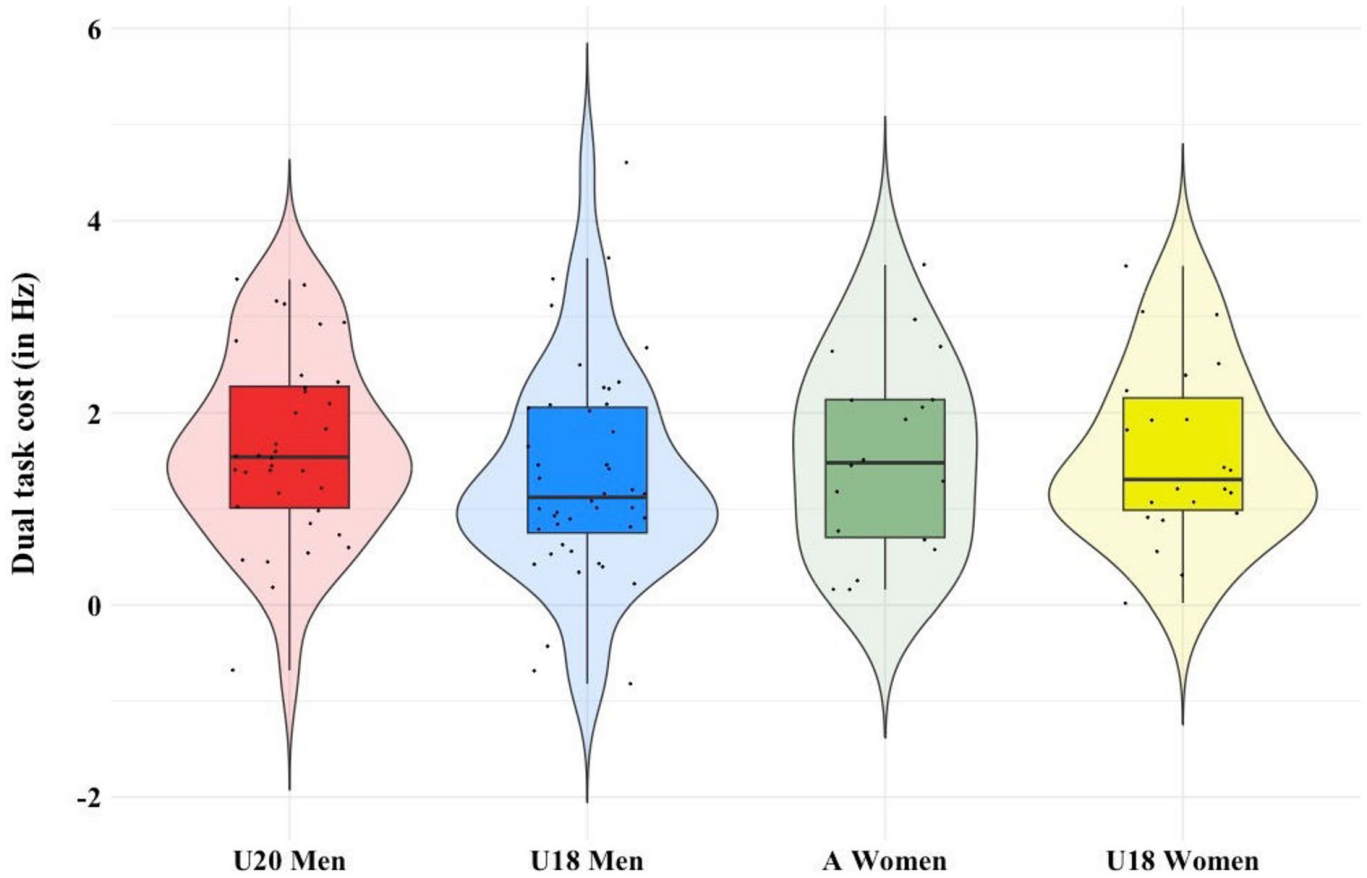
Comparison of dual task costs between ice hockey national teams.

Figure 5 depicts the difference in DTC between expertise levels. A Shapiro Wilk’s test confirmed normality of the data for DTC in men’s U20 (*W* = 0.975, *p* = 0.593), men’s U18 (*W* = 0.96, *p* = 0.1,27), women’s first team (*W* = 0.957, *p* = 0.54) and women’s U18 (*W* = 0.961, *p* = 0.502). Homogeneity of variance was confirmed, as assessed by Levene’s test for equality of variances (*p* = 0.906). A box plot indicated one outlier, which was checked, but kept in the data because it was a genuine value. The test did not indicate statistically significant differences between groups, *F*(3,116) = 1.034, *p* = 0.608.

### 3.5 DTC and cognitive parameters

For the regression, independence of residuals was established, as assessed by a Durbin-Watson statistic of 2.046. Homoscedasticity was confirmed by visual inspection of a plot of regression standardized residuals versus dependent variable and *VIF* values indicated no multicollinearity. Results of the regression model are depicted in Table 1.

**Table 1:** Multiple regression model for dual-task cost and cognitive performance.

Multiple regression model		*R*^2^ (adjusted)	*SEE* (m)		*p*-value
		0.033 (−0.039)	1.05		0.839
**Variables**	***ß***	**95% *CI***	***B***	***T***	***p*-value**
Constant		[−1.066, 6.221]	2.578	1.408	0.163
ZVT	-0,71	[−0.42, 0.025]	−0.008	−0.483	0.630
d2-R	0.016	[−0.007, 0.008]	0.000	0.120	0.905
CBT fwd.	−0.179	[−0.438, 0.081]	−0.178	−1.366	0.176
CBT bwd.	0.158	[−0.129, 0.454]	0.162	1.109	0.271
2CRT	−0.053	[−0.005, 0.003]	−0.001	−0.432	0.667
SSRT	−0.019	[−0.007, 0.006]	0.000	−0.152	0.879

SEE, standard error of estimate; β, standardized coefficient; B, unstandardized coefficient.

## 4 Discussion

The first aim of this study was to test a novel, open-skill sport-specific DT paradigm focusing on cyclical speed of the lower extremities and concurrent visuo-verbal speed-reading task in elite athletes of diverse sporting backgrounds. Three groups were distinguished in the analysis: ice hockey, other open-skill, closed-skill sport. Consistent with the results of Moreira et al. (2021), we expected all athletes to experience DTC in the paradigm. The results show significant decreases in performance from ST to DT in all three groups. The wide range in DTC highlights the heterogenous response to the DT condition. Some athletes’ performance was unaffected, whereas others displayed great performance decrements. The data seems to support that the paradigm was able to induce significant DTC, hence the first hypothesis can be confirmed. These results are in line with the review of Moreira et al. (2021), who also reported acute performance decreases under DT conditions. In one of the included studies, Fleddermann et al. (2019) employed two volleyball-specific DT tests (low and high cognitive complexity) paired with 3D-MOT. In the low-complexity task, athletes performed maximal block jumps either to the left or right, reacting to a volleyball-specific static picture. The high-complexity task also entailed maximal block jumps either to the left or right, but triggered by a volleyball-specific video, which was presented on a screen in front of the net. Results confirmed that both additional tasks induced performance decrements. Performance in the high-complexity task was worse than in the low-complexity task. It is remarkable that even most elite athletes display significant DTC in a paradigm that entails game relevant tasks, like cyclical speed of the lower body, visual perception, and verbal communication. It seems likely that athletes who display costs in this paradigm, also suffer from cognitive-motor interference in game scenarios. This might manifest in decreased speed of movement, miscommunication, or lack of communication, when cognitive and motor demands occur in parallel. Consequently, identifying the underlying causes and addressing possible weaknesses might be a promising venture to tap performance reserves.

Secondly, we wanted to check if there was a relationship between DTC and ST performance. If the DTC and ST performance were strongly correlated, one could simply discard testing of DTC and keep focus on the development of ST. However, there could be a link between both through task prioritization. Despite being instructed to not prioritize one task over the other, athletes tend to give greater priority to the motor task. Nevertheless, we expected no correlation between DTC and ST. The data analysis showed a weak positive correlation between DTC and ST. Consequently, the second hypothesis cannot be completely verified. Despite this result, we do assume sufficient independence of DTC and ST for DTC to be an important parameter to assess in athletes.

Validation of the new DT paradigm against other open-skill and closed-skill sports was the third aim of the study. Due to greater exposure to DT scenarios and the existing literature on perceptual/cognitive expertise in sports (Mann et al., 2007; Voss et al., 2010; Zhu et al., 2020), we expected differences between open-skill and closed-skill sport. Since ice hockey is an open-skill sport, differences were only expected in comparison to closed-skill sport. Furthermore, older athletes have been exposed to DT scenarios more often than younger players, hence they should show smaller performance decrements. DTC were highest in closed-skill sport, second highest in ice hockey athletes and lowest in other open-skill sports. The difference in DTC between ice hockey and open-skill sports was not significant. However, DTC in closed-skill sports were significantly higher than in ice hockey and open-skill sport athletes, respectively. Since ST performance was better in ice hockey and other open-skill sport athletes than in closed-skill sport athletes, this is even more striking. Therefore, the data seems to verify the second hypothesis. These results make it plausible, that the ability to deal with this DT situation has relevance for ice hockey and open-skill sport performance in general. These findings are in line with the work of Schaefer and Scornaienchi (2020). They compared the performance of expert and novice table tennis players in a DT paradigm entailing returning balls from a ball machine and concurrently completing a working memory task (3-back task). The researchers found that in experts, performance reduction under DT conditions was less pronounced than in novices.

The fourth aim of the study was the comparison of DTC between teams regarding the influence of age on DTC. Due the results of Moreira et al. (2021) and the assumption that older athletes should have had greater exposure to DT scenarios, we expected the women’s first team to show less DTC than the women’s U18 and the men’s U20 to show less DTC than the men’s U18. The results showed no significant differences between teams; hence the fourth hypothesis cannot be confirmed. This begs the question if greater ability to handle DT is a valid marker for superior performance in ice hockey. One possible explanation for this might be that exposure to general ice hockey training develops DT ability to a certain level, that is reached with the U18 age category or prior. Specific cognitive-motor DT training might still be able to develop this ability further. Another possibility might be that a better ability to handle DT scenarios is a selection criterion for higher performance in ice hockey that is not causally linked to DT exposure. In this case, it could be an interesting tool for early talent identification. This result is, however, limited by the fact that no direct measure of exposure to DT scenarios, like training age, was taken.

The last aim of the study was to assess the relationship between DT performance and cognitive functions to potentially identify underlying resources. Based on the resource account of Wickens (2008) as well as the studies of Casella et al. (2022), Fleddermann et al. (2019) and Moreira et al. (2021), we expected that DTC performance would rely on cognitive performance. However, no significant regression model was found that was able to predict DTC based on the cognitive performances in the ZVT, d2-R, CBT forward and backward, 2CRT and SSRT. Accordingly, the fifth hypothesis must be discarded. These results conflict with the suggestion of Moreira et al. (2021), that “individuals with high working memory capacity could optimize attentional resources for solving cognitive task while performing the motor task” and the empirical work of Fleddermann et al. (2019), who found increased performance in the processing speed and sustained attention through DT training. The authors suggested processing speed (assessed through ZVT) and sustained attention (assessed through d2-R) as an underlying resource. However, they also found no effect on memory span and letter readout. Laurin and Finez (2020) examined the interaction of working memory capacity and different cognitive tasks paired with juggling in soccer. The tasks varied in cognitive load, to test if higher working memory capacity would allow better DT performance with secondary tasks of greater difficulty. Strikingly, they found that higher working memory capacity did not appear to be beneficial but detrimental to performance under DT situations of higher complexity. Casella et al. (2022) also found improvements in cognitive performance through cognitive-motor training, but in more complex tests, the Tower of London and WISC-IV cancellation test. These tests assess more complex constructs, like planning abilities and visual search abilities, and not simply cognitive functions. Kalén et al. (2021) differentiate the cognitive constructs (1) basic cognitive functions, (2) higher cognitive functions and (3) cognitive decision-making skills and find that the latter were better at differentiating between higher-and lower skilled athletes. The authors state that “whether the advantage of specific measures for discriminating expertise levels reflects a higher level of sensitivity, a better fit of the functions and skills needed for the task, or a reflection of the combination of selection and training processes is unclear.” Scharfen and Memmert (2019) also found greater differences for cognitive skills in comparison to executive functions in elite athlete. This suggests that cognitive performance in sports, and possibly DT performance, are too complex and specific for basic or higher cognitive functions to explain great variance.

An alternative explanation for the differences in DTC might be the automatization of the employed tasks. Besides an increased capacity or resource, higher efficiency through greater degree of automatization might be an alternative adaptation to dual−/multi-tasking demands. This account assumes that processing evolves to a task-specific processing pipeline, decreasing the need for central resources (Wollesen et al., 2022). Langhanns and Müller (2018) discuss evidence for two motor regimes for repetitive movements, an automatic and a cognitively controlled regime. When a secondary cognitive task is performed simultaneously, cognitive control seems to be negatively affected, hence less automaticity could lead to greater DTC. On the one hand, this might explain the superiority of ice hockey and other open-skill sports in comparison to closed-skill sports, since the latter does not involve running, which has great similarities to the tapping task. On the other hand, there might be different levels of tapping and speed-reading automaticity within the sample of ice hockey players that could have affected the vulnerability to DTC.

This study is a valuable addition to the DT literature as it encompasses a great sample of elite athletes across multiple sports. The unique DT paradigm has enough specificity to discriminate between open-skill and closed-skill sports, but still allows assessment of a diverse spectrum of open-skill sports, that entail cyclical speed of the lower body and a concurrent visuo-verbal information processing. A limitation of this study is that only motor ST, DT and DTC were collected. This way, varying degrees of task prioritization between motor and cognitive task could not be registered. Additionally, the degree of specificity chosen to enable comparison across multiple open-skill sports, might have compromised the ability to discriminate differing ice hockey expertise levels. Lastly, athletes’ psychological state or external factors, e.g., time of testing, might be factors of influence that we were not able to control.

## 5 Conclusion

This study established the efficacy of a novel DT paradigm entailing cyclical speed of the lower body and a concurrent visuo-verbal speed-reading task in inducing DTC in a diverse array of elite athletes. DTC and ST seem to be sufficiently independent for relevancy of testing DT. The performance in this DT paradigm seems to be able to distinguish between open-skill and closed-skill sport. No differences between national teams/age groups were found. There was no relationship between DT performance and cognitive functions. From a talent identification standpoint, assessing the ability of athletes to deal with cognitive-motor interference might be a valuable addition to a performance diagnostics battery in open-skill sports. From a development perspective, athletes that display good performance in conventional speed tests like sprinting, change of direction or jumping, but still seem to lack speed in game, might benefit from testing DT ability to tap into performance reserves.

Future research should investigate DT ability of athletes over a broader age range, including cognitive ST-, DT-performance and DTC. Furthermore, specific cognitive-motor DT training might be able to develop DT ability further and should be the focus of further scientific investigations. More specific DT paradigms should be employed to better assess the relationship between expertise levels and DT ability.

## Data availability statement

The raw data supporting the conclusions of this article will be made available by the authors, without undue reservation.

## Ethics statement

The studies involving humans were approved by Ethikkommision Fachbereich 05, Goethe University Frankfurt. The studies were conducted in accordance with the local legislation and institutional requirements. Written informed consent for participation in this study was provided by the participants’ legal guardians/next of kin.

## Author contributions

MB: Conceptualization, Data curation, Formal analysis, Investigation, Methodology, Project administration, Visualization, Writing – original draft. CK: Conceptualization, Investigation, Writing – original draft. LR: Data curation, Investigation, Writing – review & editing. KZ: Conceptualization, Data curation, Funding acquisition, Methodology, Project administration, Resources, Supervision, Writing – review & editing.
